# Editorial: Resilience and Vulnerability Factors in Response to Stress

**DOI:** 10.3389/fpsyt.2019.00732

**Published:** 2019-10-17

**Authors:** Chantal Martin-Soelch, Ulrich Schnyder

**Affiliations:** ^1^IReach Lab, Unit of Clinical and Health Psychology, University of Fribourg, Fribourg, Switzerland; ^2^University of Zurich, Zurich, Switzerland

**Keywords:** resilience (psychological), vulnerability (psychological), stress, trauma, PTSD (post traumatic stress disorder)

Stress exposure is a major determinant for the development of several mental disorders (1), one of the major causes of disability worldwide (2). For both treatment and prevention of mental disorders, the identification of vulnerability and resilience factors is essential. For instance, it is still not clear which factors differentiate between individuals who will develop mental disorders, in particular, post-traumatic stress disorder (PTSD), following exposure to a potentially traumatic event from the ones who will be resilient, i.e., who may show a strong initial reaction to the event but will not suffer long-term mental health consequences and may even go on to develop post-traumatic growth (3, 4). At the moment, the interplay between vulnerability and resilience factors as well as the role of different types of stress in the development of psychopathological conditions remains unclear. Therefore, our research topic aimed to gather scientific contributions on the relationship between stress and psychopathology in general, and in particular on the field of stress-related resilience and vulnerability.

## Methodological Issues in Studying Vulnerability and Resilience Factors

To investigate vulnerability or resilience factors, prospective longitudinal designs are the methods of choice. However, these designs are costly in time and resources. Alternative designs include longitudinal, retrospective, and cross-sectional studies in victims of traumatic events or longitudinal and cross-sectional studies in so-called vulnerable populations, i.e., in individuals with an increased risk of developing mental disorders, for instance, because they are regularly exposed to stress at work (e.g., nurses in intensive care units) (Favrod et al.; Schäfer et al.), because they have experienced early-life adversity (5, 6), or because their parents suffered from a mental disorder, such as depression (7). A further methodological option is to investigate remitted PTSD patients to disentangle the effects of the disorder from potential vulnerabilities. A caveat of this design is, however, that it is not possible to differentiate between chronic, long-term sequelae from the disorder and vulnerability factors present before the onset of the disorder (8).

The majority of the articles published under this research topic report on research performed in vulnerable populations, mostly in individuals exposed to regular stress during work, including nurses (Favrod et al.; Schäfer et al.; Oe et al.), physicians (Weilenmann et al.), or students (Recabarren et al.), but also in individuals exposed to stress because of their sexual orientation or because they suffer mental disorders (Wang et al.; Tanner et al.). A narrative review article provides an overview of the current state of the research on resilience in cancer patients (Seiler and Jenewein). McGee et al. used a longitudinal design to identify potential protective factors between chronic stress and exposure to early-life adversity and health outcomes in a population of older Swiss adults. Finally, Recabarren et al. report the results of a multidimensional stress prevention program in students. Five articles report on results from studies in individuals exposed to traumatic events, including two longitudinal studies, one in assault survivors in Switzerland (Kleim et al.) and one in survivors of the Fukushima disaster in Japan. Nsabimana et al. investigated factors predicting psychological adjustment in institutionalized orphans in Rwanda using a cross-sectional design. Millon et al. report the results of a study testing autobiographical memories in survivors of sexual assault in the United States. Finally, Sun et al. tested brain network alterations in a sample of remitted PTSD patients. However, none of the published articles used a truly prospective design.

Not only designs but also validated measures are important for the investigation of vulnerability and resilience factors. Two publications addressed the question of the development and validation of questionnaires. Van der Meer et al. report the results of the Dutch and English validation of a resilience scale, the Resilience Evaluation Scale (RES), while Jacobs et al. validated the French version of the Secondary Traumatic Stress Scale.

## Vulnerability and Resilience Factors: What Do We Know?

### Vulnerability Factors

Vulnerability factors for PTSD can be categorized into pre-traumatic, peri-traumatic, and post-traumatic variables. Across all types of potentially traumatic events, variables such as female gender, low socio-economic status, or previous trauma exposure, consistently predict higher PTSD symptom levels. Type of trauma, trauma severity, and the number of traumatic event types a given person has been exposed to are the most important peri-traumatic risk factors. It has to be emphasized, however, that although statistically significant, the weighted effect sizes of all these single variables are very low. One post-traumatic variable sticks out, though, namely, lack of social support post-trauma (9, 10).

The authors of our topic have focused, on one hand, on the investigation of cognitive mechanisms involved in the development of PTSD and, on the other hand, on the identification of vulnerability factors specific to the context of the stress experience, extending therefore the state of the research. With regard to cognitive processes, Kleim et al. evidenced in a sample of assault survivors in Switzerland the role of specific linguistic markers, showing that more cognitive elaboration is associated with lower risk for PTSD. Millon et al. reported that autobiographic memories of stressful events are stronger in women with than in women without experience of sexual violence and that the strengths of these memories strongly correlated with traumatic cognitions, ruminative thoughts, and psychopathological scores irrespective of the presence of PTSD. Changes in neural connectivity also differentiated healthy individuals from PTSD and remitted PTSD, suggesting that specific neural functioning could be a vulnerability factor for PTSD (Sun et al.).

Context-specific vulnerability factors were evidenced in traumatized child samples, with Nsabimana et al. reporting that being institutionalized in an orphanage and having at least one living parent is a vulnerability factor for reduced self-esteem and increased externalizing behaviors. Further, Oe et al. identified important factors for the parent’s recognition of school bullying in survivors of the Fukushima disaster (Oe et al.). Both studies have a specific cultural context, as parents in Rwanda place their children in orphanages out of poverty or in the hope that they will get a better education (Nsabimana et al.); and in Japan, displaced survivors of the Fukushima disaster are often victims of stigmatization (Oe et al.). Context-specific vulnerability factors were also reported in vulnerable samples. For instance, Favrod et al. showed that symptoms of secondary post-traumatic stress in nursing staff are specific to the work environment, with higher scores reported in nurses working in neonatal intensive care than in midwives and with more work-related traumatic stressors reported in the former group. In contrast, a cross-cultural study indicates that midwives working in Switzerland have higher symptom levels of psychological distress and secondary traumatic stress than midwives working in Japan (Oe et al.). These findings stress again the importance of the cultural context. In individuals suffering mental illness in Switzerland, fear of negative evaluation, fatigue, concentration problems, negative alterations in mood, and dissociative symptoms showed the strongest negative association with recovery from functional impairment after 18 months (Tanner et al.), suggesting that specific psychiatric symptoms and stigmatization (or fear of stigmatization) represent vulnerability factors in this sample.

### Resilience Factors

The best documented psychological protective factors include perceived social support [see Refs. (11, 12) for review], sense of coherence (SOC) (13, 14), self-efficacy, and sense of mastery (15–17). It is therefore not surprising that three articles published in this research topic examined the protective effect of SOC. These studies evidenced a strongly predictive effect of SOC on general mental health in nurses of intensive care units (Schäfer et al.), as well as a stress-mediating effect in older adults (McGee et al.), and SOC was identified as a resilience factor in cancer patients (Seiler and Jenewein).

In addition to the well-documented resilience factors, the contributions to our research topic investigated new and promising variables, including adaptive emotion regulation strategies and empathy (Weilenmann et al.), as well as mindfulness abilities (Wang et al.). This last study used an interesting approach, creating an index for psychosocial resources based on the scores of 14 general factors related to resilience. From those, mindful attention, purpose in life, non-rumination, positive affect, vitality, and positive relationships with others stuck out as the most protective ones (Wang et al.).

## Conclusion and Clinical Implications

Our research topic also points out the necessity to develop well-validated measures of stress, resilience, and vulnerability, in particular, culturally adapted measures to stimulate research outside of the industrialized countries. Further, none of the presented studies used a prospective longitudinal design in vulnerable individuals, which would be the method of choice to identify vulnerability and resilience factors. This should be the focus of future studies and become a priority for funding agencies.

In addition, this research topic highlights the role of well-documented resilience factors, including perceived social support and SOC, to mediate the effects of stress on mental health in vulnerable individuals. In addition, mindfulness abilities, successful emotion regulation strategies, and positive affect, among others, were identified as potentially promising protective factors. The identification of resilience and protective factors is in turn relevant for the development of specific clinical prevention and intervention programs. This is well illustrated by the results of a preventive stress prevention program (Ge-D-Stress) in university students that successfully increased quality of life and resilience factors and was able to decrease psychological symptoms as well by activating several psychosocial resources (Recabarren et al.). However, the majority of the published works in this research topic investigated vulnerability factors. Interestingly, they focused on the role of cognitive and neural processes as well as of context-specific factors, for instance, related to the work environment or the cultural context. A better understanding of specific vulnerability factors might allow us to detect individuals at risk in specific populations in order to provide tailored preventive or clinical interventions, such as the Ge-D-Stress program.

## Author Contributions

Both authors contributed in writing of the editorial.

## Conflict of Interest

The authors declare that the research was conducted in the absence of any commercial or financial relationships that could be construed as a potential conflict of interest.
